# Identification and characterization of a novel ISG15-ubiquitin mixed chain and its role in regulating protein homeostasis

**DOI:** 10.1038/srep12704

**Published:** 2015-07-30

**Authors:** Jun-Bao Fan, Kei-lchiro Arimoto, Khatereh Motamedchaboki, Ming Yan, Dieter A. Wolf, Dong-Er Zhang

**Affiliations:** 1 Moores UCSD Cancer Center, University of California San Diego, La Jolla CA, 92093, USA; 2 NCI Cancer Center Proteomics Facility, Sanford Burnham Prebys Medical Discovery Institute, La Jolla, CA 92037, USA; 3 Tumor Initiation & Maintenance Program, Sanford Burnham Prebys Medical Discovery Institute, 10901 North Torrey Pines Road, La Jolla, CA 92037, USA; 4 San Diego Center for Systems Biology, La Jolla, CA 92093-0375, USA; 5 Department of Pathology and Division of Biological Sciences, University of California San Diego, La Jolla CA, 92093, USA

## Abstract

As a ubiquitin-like modifier, ISG15 is conjugated to many cellular proteins in a process termed protein ISGylation. However, the crosstalk between protein ISGylation and the ubiquitin proteasome system is not fully understood. Here, we report that cellular ubiquitin is a substrate of ISG15 and Lys 29 on ubiquitin is the major ISG15 acceptor site. Using a model substrate, we demonstrate that ISG15 can modify ubiquitin, which is immobilized on its substrate, to form ISG15-ubiquitin mixed chains. Furthermore, our results indicate that ISG15-ubiquitin mixed chains do not serve as degradation signals for a ubiquitin fusion degradation substrate. Accordingly, an ISG15-ubiquitin fusion protein, which mimics an ISG15-ubiquitin mixed chain, negatively regulates cellular turnover of ubiquitylated proteins. In addition, ISG15-ubiquitin mixed chains, which are detectable on endogenously ubiquitylated proteins, dampen cellular turnover of these proteins. Thus, our studies unveil an unanticipated interplay between two protein modification systems and highlight its role in coordinating protein homeostasis.

Cellular proteins degraded by proteasomes are often linked to a polyubiquitin chain, in which the C-terminal carboxyl group of a ubiquitin is coupled to a lysine of the proximal ubiquitin through an isopeptide bond to the ε-amino group of the lysine^1^,^2^,^3^,^4^. Ubiquitin contains seven lysines (Lys6, Lys11, Lys27, Lys29, Lys33, Lys48 and Lys63), all of which can be acceptors for the chain formation^5^. Emerging evidence suggests a topological diversity and complexity to ubiquitin chain structure and biological functions. It is well documented that Lys48-linked chains guide substrate degradation by the proteasome, while Lys63-linked chains are involved in several cellular processes such as signal transduction and DNA repair^6^,^7^,^8^. Relatively less is known about the precise function of chains that are linked through Lys6, Lys11, Lys27, Lys29 and Lys33. Moreover, previous studies reported the presence of several types of “atypical” ubiquitin chains^9^, including linear ubiquitin chains, in which the ubiquitin moieties are linked to each other in a ‘head-to-tail’ manner^10^,^11^,^12^, and multiply branched chains, in which two (or perhaps even more) ubiquitin moieties are anchored to distinct lysine residues in a single moiety^13^,^14^,^15^.

Other than ubiquitin self-modification to form ubiquitin chains, little is known about other types of modifications on ubiquitin and their biological functions. SUMO was reported as a ubiquitin-like protein that crosstalks with ubiquitin, and major attention has been paid to the incorporation of ubiquitin to SUMO chains^16^,^17^,^18^. A specific example is the promyelocytic leukemia (PML) protein, which is initially polySUMOylated. The SUMO chains then recruit the ubiquitin ligase RING finger protein 4 (RNF4), by which the ubiquitin moieties are added to the SUMO chain and result in proteasomal degradation of PML. In addition, SUMO also modifies ubiquitin at Lys63, however the biological significance of mixed SUMO-ubiquitin chains is still unknown^19^. Recently, modification of ubiquitin or its substrates by NEDD8 under stress conditions has been reported^20^,^21^,^22^,^23^. NEDD8 modifies ubiquitin at Lys 48 and these NEDD8-ubiquitin heterologous chains are recognized essentially like ubiquitin chains^21^. Another ubiquitin-like protein, FAT10, has been reported as a degradation signal when it is fused to the N-terminus of long-lived proteins^24^,^25^. However the possible modification of ubiquitin by FAT10 has not been reported. Thus our understanding of the crosstalk between ubiquitin-like proteins and the UPS is in its infancy, and the evidence of the physiological importance of these modifications is largely absent.

ISG15 is the first identified ubiquitin-like modifier, which can covalently conjugate to cellular proteins^26^,^27^. Its expression is strongly upregulated by type I interferon (IFN)^28^. As with the ubiquitin system, there are a series of distinct enzymes involved in the process of protein ISGylation, including ISG15 activating enzyme (E1)-UBE1L^29^,^30^, conjugating enzyme (E2)-UBCH8^31^,^32^, protein ligase (E3)^33^,^34^,^35^,^36^, and ISG15 specific protease USP18^37^,^38^, as well as some viral proteins^39^. To understand the function of ISG15 conjugation, major efforts have been spent to identify ISGylated proteins under physiological conditions^40^ and in HeLa cells expressing the ISG15 conjugation system^35^,^41^. Several substrates of ISG15 and their roles in cellular function have been reported previously^34^,^42^,^43^,^44^,^45^,^46^,^47^.

As a ubiquitin-like modifier, the mature form of ISG15 contains two ubiquitin-like domains and a C-terminus ending with the amino acid sequence ‘Leu Arg Leu Arg Gly Gly’ (LRLRGG). This motif is identical to the C-terminus of mature ubiquitin. Relative to what we have learned about ubiquitin and the ubiquitin-like modifiers SUMOs and NEDD8, little is known about protein modification by ISG15. It has been shown that proteasome inhibitors modulate the level of cellular ISGylated proteins^48^ and that increased protein ISGylation is associated with decreased levels of polyubiquitylated proteins^49^. Meanwhile, free ISG15 binds to NEDD4, and blocks NEDD4-mediated protein ubiquitylation^50^. Furthermore, protein modification by ISG15 is reported to reduce the turnover of cellular ubiquitylated proteins^51^. However, similar levels of polyubiquitylated proteins were detected in ISGylation deficient cells as in wild-type cells^52^. These findings suggest that the apparent antagonistic relationship between ISGylation and ubiquitylation might be restricted to specific cell conditions. On the other hand, the molecular networks coupling the ISG15 conjugation system to the UPS are not fully understood. Here we report the formation of ISG15-ubiquitin mixed chains and describe their role in regulating the turnover of ubiquitylated proteins.

## Results

### ISG15 Conjugates to Ubiquitin

**Figure 1:**
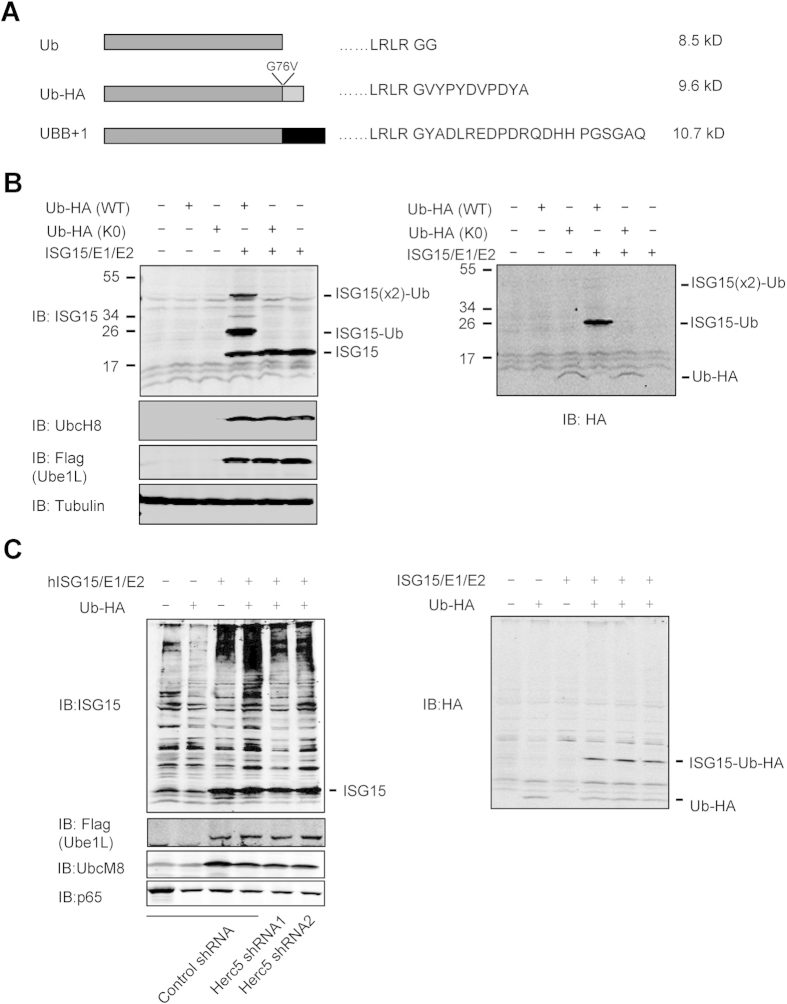
Ubiquitin is a substrate of ISG15. (**A**) Different constructs expressing ubiquitin were generated for examination of ISGylation on ubiquitin. The expected molecular weight of these proteins was determined using the ExPASy-ProtParam tool. For Ub-HA, Gly76 was mutated to valine to avoid proteolytic removal of the HA tag in the cells. (**B**) Ub-HA is a substrate of ISG15. 293T cells were transfected with different constructs as indicated. Cell lysates were subjected to SDS-PAGE and detected by antibodies as indicated. (**C**) ISGylation of ubiquitin is not sensitive to cellular disruption of HERC5. 293T cells stably expressing control or HERC5 shRNA were transfected with different constructs as indicated. Cell lysates were subjected to SDS-PAGE and detected by antibodies as indicated.

In a proteomic study to identify ISG15 conjugated proteins, we found that ISG15 has the potential of modifying ubiquitin^40^. We further studied whether ubiquitin was a substrate of ISGylation in cells over-expressing ISGylation components and ubiquitin. To avoid possible interference from ubiquitylation of ISG15 (~17 kDa), we used C-terminally HA-tagged ubiquitin (Ub-HA, ~8 kDa) and UBB+1 (~10 kDa) in this study (Fig. 1A). UBB+1 is a natural ubiquitin variant identified from the brain of patients with Alzheimer’s Disease^53^. ISGylated ubiquitin was detected after co-expression of Ub-HA and ISG15 conjugating components (HIS-ISG15, Ube1L, and UbcH8) in 293T cells (Fig. 1B). Two ISG15 modified forms of ubiquitin were detected. The major modified form is ~25 kDa, which equals to the molecular weight of one ISG15 and one ubiquitin. Another ISG15 modified form is ~40 kDa, which is relatively weak in an anti-ubiquitin blot (right panel of Fig. 1B) and more readily detected by immunoprecipitation of ubiquitin (Figure S1). This ~40 kDa band most likely contains two molecules of ISG15 conjugated to two lysines on one ubiquitin molecule (Figure S1).

Given that human HERC5-mediated ISG15 conjugation broadly targets newly synthesized proteins and occurs cotranslationally in cells^42^, we further investigated whether ISGylation of ubiquitin occurs cotranslationally. Our results suggested that disruption of HERC5 in 293T cells does not obviously affect ISGylation of ubiquitin (Fig. 1C and Figure S1B). Therefore, ISGylation of ubiquitin may not be strictly restricted to a co-translational process. In the case of UBB+1, similar results were obtained in both 293T and HeLa cells (Figure S2A and S2B). To demonstrate that cellular endogenous ISG15 can conjugate to ubiquitin, UBB+1 was stably expressed in WT and Ube1L KO mouse embryonic fibroblasts (MEFs). ISG15 conjugated UBB+1 was only detected in WT MEFs but not in Ube1L KO MEFs (Figure S2C). Furthermore, when lysine free UBB+1 (UBB+1 K0) was used, no ISGylated UBB+1 was detected, although UBB+1 K0 is much more stable than WT UBB+1 (Figure S2C). These results show that ISG15 directly modifies ubiquitin.

**Figure 2:**
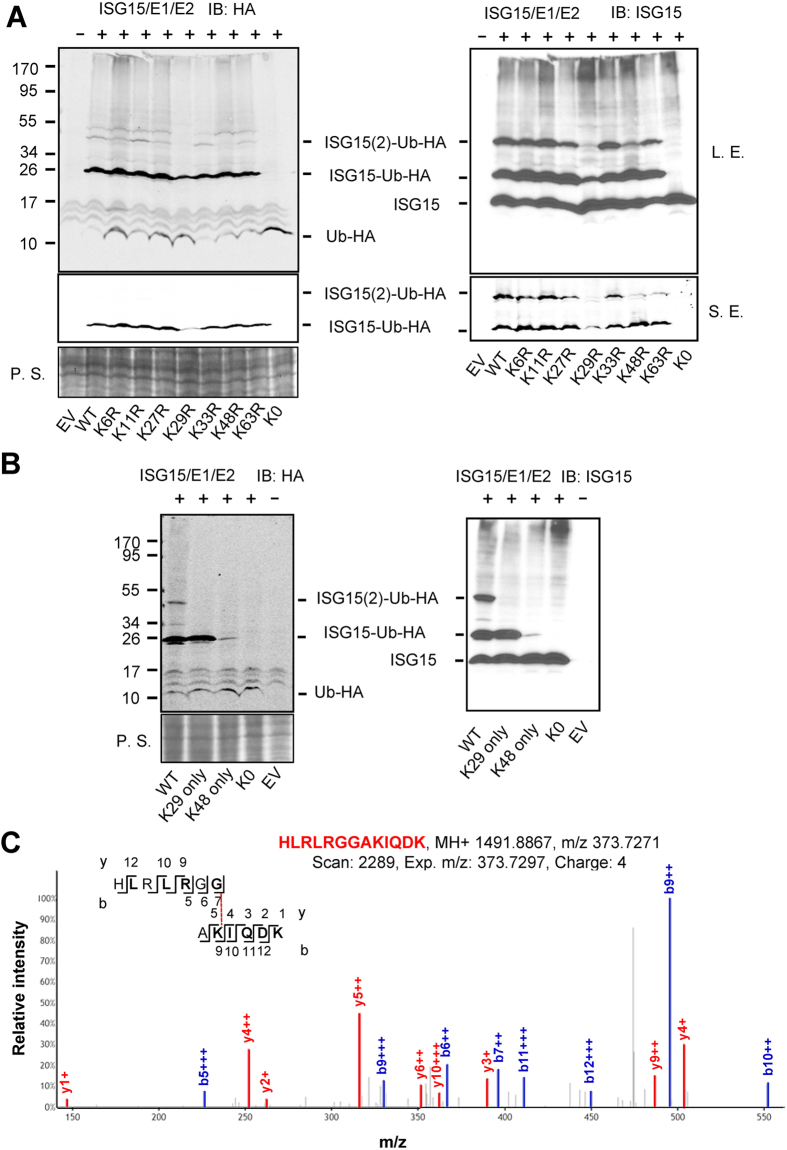
ISG15 is primarily conjugated to Lys29 of ubiquitin. (**A**) ISGylation of ubiquitin mutants with each lysine mutated to arginine (termed KXR). (**B**) ISGylation of ubiquitin mutants with single lysines (termed KX-only). 293T cells were transfected with different constructs as indicated. Cell lysates were subjected to SDS-PAGE. The blot was probed with HA and ISG15 antibodies. L. E., long exposure; S. E., short exposure. (**C**) Tandem mass spectrum of an ISG15 C-terminal peptide linked to lysine 29 of ubiquitin. The b- and y-type product ions are marked and labeled along the peptide sequence shown on top of the spectrum. P. S. stands for ponceau staining.

To identify the preferred ISGylation sites on ubiquitin, we mutated each lysine on ubiquitin individually to arginine (termed KXR). Upon co-expression of ubiquitin mutants and ISG15 conjugation components, the K29R mutant showed a decreased signal at ~25 kDa (ISG15-Ub-HA) compared to other mutants. Meanwhile, both the K29R and the K48R mutants showed a decrease in the ~40 kDa signal (ISG15 × 2-Ub-HA) (Fig. 2A). These data suggest that Lys 29 of ubiquitin is the major site for ISGylation, while Lys 48 is secondary site that can be ISGylated to some degree. No signal equivalent to ISG15 dimers (i.e. ISGylated ISG15) was detected, which supports the conclusion that ISG15 × 2-Ub-HA represents a ubiquitin ISGylated on two different lysines. We also restored lysines 29 and 48 in ubiquitin K0 (termed KX-only). Consistent with the above results, Ub-K29-only gave an ISGylation pattern similar to wild-type ubiquitin with a prominent band at ~25 kDa but not at ~40 kDa. Ub-K48-only showed weak ISGylation resulting in the ~25 kDa band (Fig. 2B). When ISG15 modified ubiquitin was isolated from cells and subjected to liquid chromatography and tandem mass spectrometry, Lys 29 of ubiquitin was unequivocally identified as the acceptor lysine of ISG15 (Figure S3 and 2C). Together, these results demonstrate that ubiquitin is an ISGylation substrate. Furthermore, Lys 29 of ubiquitin is the major site of ISGylation.

### ISG15 can be conjugated to ubiquitin attached to a model substrate protein

**Figure 3:**
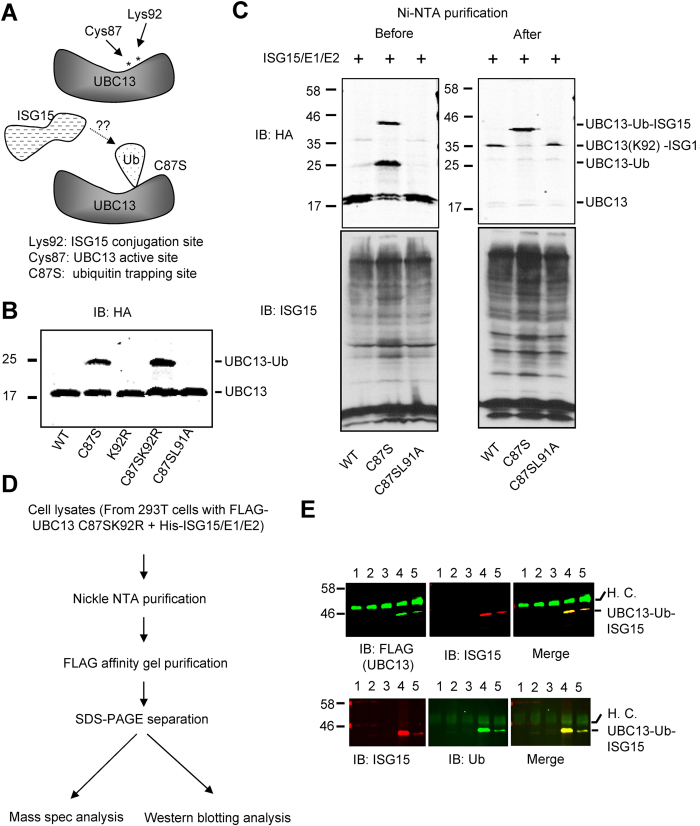
ISG15 is conjugated to ubiquitin on a ubiquitylated model substrate. (**A**) Cartoon diagram of ubiquitin trapping site on UBC13. UBC13 mutant (UBC13 C87S) can form a covalent bond with ubiquitin through an ester bond between Ser 87 of UBC13 C87S and Gly 76 at the C-terminus of ubiquitin. (**B**) Endogenous ubiquitin modifies UBC13 mutants in 293T cells. (**C**) ISG15 conjugates to UBC13 trapped ubiquitin in 293T cells. (**D**) Workflow for isolating the UBC13-Ub-ISG15 triple complex from cell extracts. (**E**) Confirmation of the isolated UBC13-Ub-ISG15 triple complex with specific antibodies. Lanes 1–5, proteins immobilized on beads after incubating the FLAG affinity gel with different fractions from Nickle NTA purification: 1, flow through, 2–5, elutes with 10 mM, 40 mM, 350 mM, or 500 mM imidazole, respectively. In (**B**–**E**), 293T cells were transfected with different constructs as indicated. Protein samples were subjected to SDS-PAGE and electroblotted and detected by antibodies as indicated.

Molecular trapping has been successfully used in structural biology to prepare crystals of large molecular complexes. A UBC13 mutant (UBC13 C87S), which can form a covalent bond between ubiquitin and UBC13, was used previously to determine the structure of the UBC13-ubiquitin-Ube1V heterotrimer^54^. This stable complex is formed through an ester bond between Ser 87 of UBC13 C87S and Gly 76 at the C-terminus of ubiquitin. Here, we used the same approach to study whether ISG15 can be conjugated to ubiquitylated proteins using UBC13-ubiquitin as a model substrate (Fig. 3A).

We first confirmed that the UBC13 C87S mutant was stably mono-ubiquitylated by endogenous ubiquitin, while wild-type UBC13 and UBC13 C87S/L91A were unable to trap ubiquitin (Fig. 3B). We also showed that ISG15 was not trapped on UBC13 C87S (Figure S4A). These data show that UBC13 C87S specifically traps ubiquitin and this complex can be used as a model ubiquitylated protein. Furthermore, Lys 92 is the only known ISG15 modification site of UBC13^47^,^55^. Mutation of this residue (UBC13 C87S/K92R) did not disrupt ubiquitin trapping (Fig. 3B). To demonstrate ISGylation of ubiquitin attached to a substrate protein, UBC13 or its mutants were co-expressed with ISGylation components as shown in Fig. 3C. We detected a ~40 kDa band only in cells that also had UBC13-ubiquitin (UBC13 C87S and UBC13 C87S/K92R) (left panel of Fig. 3C and Figure S4B). 40 kDa corresponds to the approximate molecular weight of UBC13-Ub-ISG15 (UBC13–17 kDa, Ub-8 kDa, ISG15–17 kDa). After Ni-NTA purification of HIS-ISG15 conjugated protein, the ~40 kDa band was enriched in the samples derived from cells expressing UBC13 C87S. In contrast, a ~35 kDa band, which represents UBC13 modified with ISG15 on Lys 92, was observed in samples from cells expressing UBC13 and UBC13 C87S/L91A, which are unable to trap ubiquitin (right panel of Fig. 3C). To rule out that direct ISGylation of UBC13 in the ubiquitin trapping assay impacts our interpretations, we also examined the modification of UBC13 C87S/K92R. A similar band of ~40 kDa was detected in UBC13 C87S/K92R expressing samples as in UBC13 C87S expressing samples (Figure S4B). Based on the structural information of the UBC13-ubiquitin-UBE1V heterotrimer, we speculate that strong mono-ubiquitylation at Ser87 may spatially block the adjacent ISGylation on Lys 92. These results indicate that the ~40 kDa band corresponds to ISGylated ubiquitin, which is covalently attached to UBC13 C87S or UBC13 C87S/K92R.

Lastly, to confirm that the 40 kDa band is indeed a UBC13-Ub-ISG15 complex, protein extracts were prepared from cells expressing FLAG-UBC13 C87S/K92R and the ISGylation system. The ISGylated UBC13 C87S/K92R was isolated by a two-step purification method as indicated in Fig. 3D. Western blotting showed that the 40 kDa band was recognized by antibodies to ISG15, ubiquitin, and FLAG (for UBC13) (Fig. 3E). It is worth noting that the ISG15 and ubiquitin antibodies are specific for ISG15 and ubiquitin, respectively (data not shown). We also examined proteins in the 40 kDa band by mass spectrometry. Spectra representing all three proteins were detected with coverages of 66% (UBC13), 68% (ISG15), and 61% (ubiquitin) (Figure S5).

### ISG15-ubiquitin mixed chains are not degradation signals

**Figure 4:**
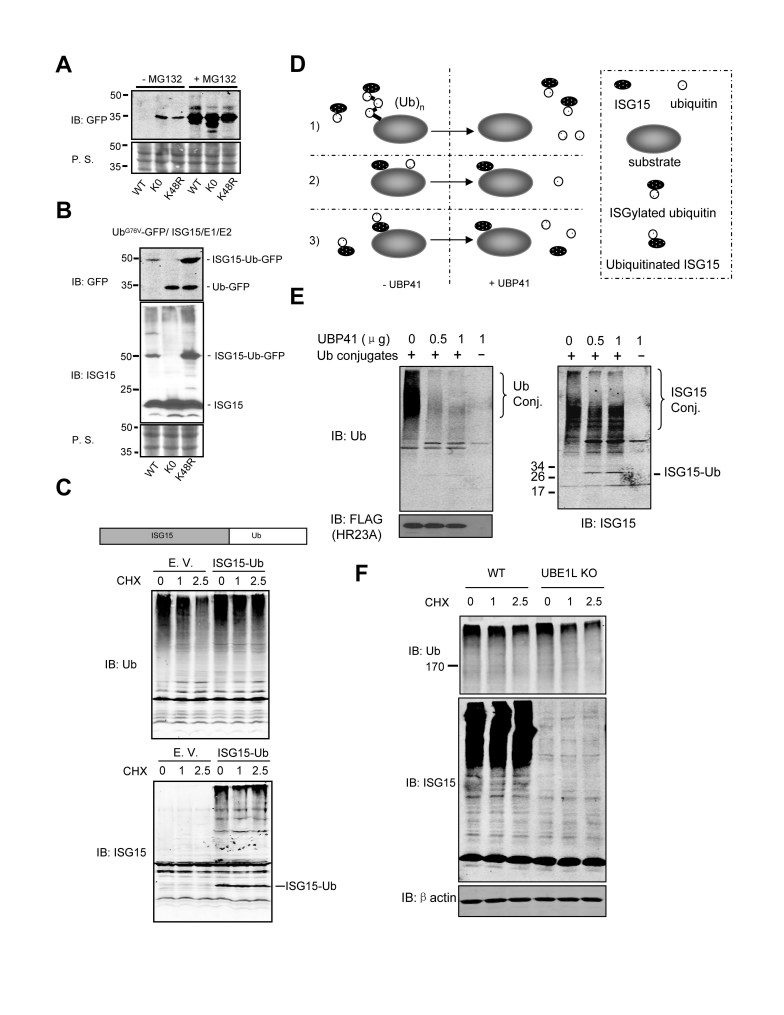
ISG15-ubiquitin mixed chains are not degradation signals but regulate the turnover of ubiquitylated proteins. (**A**) A UFD substrate, Ub^G76V^-GFP, is regulated by proteasomal degradation. 293T cells were transfected with different constructs and treated with 10 μM MG132 as indicated. Cell lysates were subjected to SDS-PAGE and with antibodies as indicated. (**B**) ISG15 conjugated Ub^G76V^-GFP is more resistant to proteasomal degradation. 293T cells were transfected with different constructs. Cell lysates were subjected to SDS-PAGE and with antibodies as indicated. (**C**) Linear ISG15-ubiquitin fusion protein affects the turnover of cellular ubiquitylated proteins. 293T cells were transfected with control vectors or vectors expressing linear ISG15-ubiquitin fusion proteins. After 40 h, cells were treated with cycloheximide (CHX, 50 μg/mL) for various periods and cellular ubiquitylated proteins were detected by immunoblotting. (**D**) Possible modes of ISG15-ubiquitin conjugation. UBP41 is a ubiquitin specific protease that does not cleave ISG15 conjugates. (**E**) Detection of ISG15-ubiquitin conjugates by chain disassembly assay. Cellular ubiquitylated proteins were isolated from MEFs by ubiquitin binding protein HR23A (with FLAG tag). Proteins purified by FLAG affinity chromatography were incubated with GST-UBP41. Protein samples were mixed with SDS sample loading buffer and probed with ISG15 antibody and ubiquitin antibody. (**F**) Protein ISGylation affects the turnover of cellular ubiquitylated proteins. MEFs were treated with type I IFN (1000 U/mL) for 24 hours followed by the addition of CHX (50 μg/mL) for the indicated periods. The decay of cellular ISGylated and ubiquitylated proteins was measured by immunoblotting with ISG15 and ubiquitin antibodies. P. S. stands for ponceau staining.

To investigate the effect of ISG15-ubiquitin mixed chains on cellular protein homeostasis, we used Ub^G76V^-GFP as a model substrate to examine whether ISG15-ubiquitin mixed chains affect cellular protein degradation by the proteasome. Wild-type Ub^G76V^-GFP (WT) is degraded rapidly by the UPS, while mutation of the lysines of the ubiquitin portion (lysine free or K48R) partially stabilized the protein (Fig. 4A). The proteasome inhibitor MG132 increased accumulation of all forms of Ub^G76V^-GFP as reported previously^56^. In the presence of ISG15 conjugation components, ISG15-linked Ub^G76V^-GFP (WT) was detected, but not the unconjugated form (Fig. 4B). These results indicate that ISG15-conjugated Ub^G76V^-GFP (WT) is more resistant to proteasomal degradation than the unconjugated form. Furthermore, ISGylation was not detectable on Ub^K0,G76V^-GFP although it is much more stable than Ub^G76V^-GFP. For Ub^K48R,G76V^-GFP, we detected much more of the ISG15-conjugated form than the unconjugated form (Fig. 4B), supporting ISGylation at Lys29 of ubiquitin (Fig. 2). These data suggest that ISG15-ubiquitin mixed chains are not degradation signals. This conclusion is strengthened by our observation that expression of a linear ISG15-ubiquitin fusion protein, which mimics ISG15 conjugated to ubiquitin, reduced cellular turnover of ubiquitylated proteins (Fig. 4C).

### ISG15 is conjugated to cellular ubiquitylated proteins

ISG15 may be conjugated to endogenous ubiquitylated proteins in three different patterns: 1) ISG15 may be conjugated to ubiquitin directly, thereby attaching to ubiquitylated proteins forming an ISG15-ubiquitin mixed chain, 2) ISG15 and ubiquitin may be conjugated to the same substrate through different sites, 3) ISG15 itself might be ubiquitylated (Fig. 4D). To further study the incorporation of ISG15 into cellular ubiquitin chains, we examined whether ISG15 was present in purified ubiquitylated proteins. Following a previously established method^57^, we used ubiquitin binding protein HR23A to isolate ubiquitylated proteins from lysate of WT and Ube1L KO MEFs. ISG15 conjugates were readily detected in HR23A-bound ubiquitylated proteins (Fig. 4E). These results demonstrated the presence of ISG15 in the pool of ubiquitylated proteins.

We used a ubiquitin chain disassembly assay to verify that ISG15 directly modifies ubiquitin on endogenous cellular ubiquitylated proteins (Fig. 4D,E). We previously showed that UBP41 was a ubiquitin-specific protease and was inactive toward ISG15 conjugates^38^. Digestion of ubiquitin conjugates containing ISG15-ubiquitin mixed chains with UBP41 should reduce the signal for high molecular weight ubiquitin and ISG15 species while liberating ISG15- conjugated ubiquitin (~25 kDa); which reflects possibility 1 in Fig. 4D. As shown in Fig. 4E, UBP41 digestion removed ubiquitin from ubiquitylated proteins and also decreased the intensity of ISGylated proteins, especially for a pool of ubiquitylated proteins with high molecular weight. In addition, a new band of ~25 kDa was detected in both Ub and ISG15 Western blots (Fig. 4E). These data indicate that ISG15 can modify cellular ubiquitylated proteins by forming ISG15-ubiquitin mixed chains. Meanwhile, a pool of ubiquitylated proteins with high molecular weight showed slower turnover in the presence of protein ISGylation (Fig. 4F), which is likely due to their modification with ISG15-ubiquitin mixed chains. These data confirmed that ISG15-ubiquitin mixed chains form on a portion of endogenous ubiquitylated proteins and negatively regulate cellular turnover of these proteins.

## Discussion

In the present study, we report that ubiquitin is a substrate of ISG15. By conjugating to ubiquitin, ISG15 incorporates into ubiquitin chains and negatively regulates the turnover of ubiquitylated proteins. ISG15-targeted proteins have been identified with proteomic methods^35^,^40^,^41^. The 293T conjugation system is a potent tool for validating the substrates identified by proteomic methods. In this system, ISG15 modifies only a very small portion of a given protein, which can only be detected after affinity enrichment^40^,^41^,^46^. We showed in this report that, unlike previously identified ISG15 substrates, ubiquitin with a blocked C-terminus was a much more efficient substrate in the 293T conjugation system.

We identified Lys 29 of ubiquitin as the major conjugation site for ISG15, which is different from the modification site of ubiquitin by either NEDD8 or SUMO. Lys 48 of ubiquitin is also targeted by ISG15. Lys 29-linked ubiquitin chains have not been extensively studied, although a few examples were reported previously. In the case of ubiquitin fusion degradation (UFD) substrates, both Lys 29 and Lys 48 were reported as important anchors for ubiquitin chain formation to target UFD substrates for proteasomal degradation^58^. Lys 29-linked polyubiquitin chains were also reported as a degradation signal for lysosomal degradation of Deltex, which was mediated by Itch/AIP4, two regulators involved in the Notch signaling pathway^59^. Likewise, TRAF7 promotes Lys 29-linked polyubiquitylation of NEMO and p65, which results in lysosomal degradation of both proteins and perturbed NF-κB signaling^60^. Since ISG15-ubiquitin mixed chains are not signals for proteasomal targeting, ISG15 may generally interfere with processes controlled by Lys 29-linked polyubiquitin chains, a possibility that remains to be addressed in future studies.

Relying on cycloheximide chase, Wood *et al*. have reported that ISGylation affects the turnover of ubiquitylated proteins in Ataxia Telangiectasia cells^51^. Our study indicates that ISG15-ubiquitin mixed chains exist on a fraction of ubiquitylated proteins and contribute to their slower turnover, a finding that supports the previous report and provides a novel molecular mechanism for regulating the UPS by protein ISGylation. However, reliable methods are not available to quantify the abundance of ISG15-ubiquitin chains in the highly heterogeneous pools of ubiquitin chains, partially because the two proteins share the same amino acid sequence at the C terminus that is essential for their function. Moreover, ISG15 conjugation broadly targets newly synthesized proteins and occurs cotranslationally in cells^42^, which makes it difficult to reconstitute the ISG15 conjugation system in test tubes in the absence of active protein synthesis. All the above could be of great interest in the future for more detailed insights into the ISG15-mediated regulation of the UPS system and its biological functions.

In summary, we observed strong ISGylation of ubiquitin in co-expression systems, however endogenous ISG15-ubiquitin mixed chains seem to be only restricted to certain cellular proteins. To our knowledge, this is the first report of the formation of ISG15-ubiquitin mixed chains and their effect on UPS function. In addition to blocking polyubiquitin chains formation, protein ISGylation can regulate the UPS system by other means such as competition for E1/E2/E3s ubiquitylation enzymes^50^ and for substrate lysines^49^. In combination, these different arms convey a general inhibitory effect on UPS function, which may lead to multiple downstream effects, including the activation of the autophagy pathway^61^, the formation of cellular inclusions^51^, and inflammatory cytokine production (Fan *et al*. manuscript in preparation). This study sheds new light on the complexity and heterogeneity of ubiquitin chains *in vivo*, especially under IFN-related stress conditions such as pathogen infection, neurodegeneration, and cancer development.

## Materials and Methods

### Plasmid construction and mutagenesis

Ubiquitin cDNA, with all lysines mutated to arginines, was kindly provided by Dr. Michele Pagano (New York University, NY). Plasmids expressing C-terminus HA-tagged ubiquitin and its mutants were generated by PCR using human ubiquitin and its mutants as the templates. Plasmids expressing UBB+1 (pcDNA 3.1-UBB+1 and MSCV-UBB+1) and its mutant were generated by PCR using human ubiquitin as a template. Plasmids expressing Ub^G76V^-GFP and its mutants were generated according to a previous report^56^. Plasmids expressing His-ISG15, UbcH8, and Ube1L have been described previously^34^,^47^. FLAG-Ube1L cDNA was cloned into pMSCV-puro (Clontech). All the constructs were confirmed by DNA sequencing. Plasmids expressing UBP41 (as GST fusion) construct was provided by Dr. R. Baker (Australian National University, Canberra). Vectors for expressing shHERC5 were generated by subcloning human HERC5 target oligonucleotide (#1: 5′-GGACTAGACAATCAGAAAGTT-3′; #2: 5′-CCACCACACCACAGATTGT-3′) into pSUPER.retro.puro vector^35^.

### Cell culture, transfections and infection

HEK293T cells were cultured in Dulbecco’s modified Eagle’s medium (DMEM) (Invitrogen, Carlsbad, CA) with 10% bovine calf serum (BCS) (HyClone, Logan, UT), 2 mM L-glutamine (Invitrogen) and Penicillin/streptomycin (100 U/ml, Invitrogen). 293T Cells were transfected using polyethylenimine (PEI) reagent^62^. HeLa cells and MEFs were cultured in DMEM with 10% fetal bovine serum (FBS) (HyClone), 2 mM L-glutamine, and Penicillin/streptomycin (100 U/ml). For UBB+1 stably expressing MEFs, 293T cells were transfected with MSCV-UBB+1 and ecopac (pIK6.1MCV.ecopac.UTd) using PEI reagent. Retroviruses from the culture medium of these cells were infected to MEFs and stable infected cells were selected by hygromycin (200 μg/ml). For cells stably expressing shHERC5, 293T cells were transfected by vectors expressing control shRNA or HERC5 shRNA and selected by puromycin (1 μg/ml).

### Proteins, antibodies and Western blot analyses

Universal type I IFN was purchased from PBL biomedical laboratories (Piscataway, NJ). Antibodies against FLAG (Sigma-aldrich), Tubulin (Sigma-Aldrich), HA (Covance, Denver, PA & Santa Cruz), β-actin (Sigma-Aldrich), UBB+1 (Millipore), GFP (Invitrogen), FK1 (Enzo life Sciences) and ubiquitin (eBioscience) were purchased from the respective manufacturers. Proteins were electroblotted onto nitrocellulose membranes (HyBond, Amersham Biosciences Inc). Rabbit anti-mouse ISG15 polyclonal antibodies have been described previously (Malakhova *et al*., 2003). Armenian hamster ISG15 monoclonal antibody is a kind gift from Dr. Debra Lenschow (Washington University, MA). Fluorophore conjugated secondary antibodies (Li-Cor) or horseradish peroxidase (HRP) conjugated secondary antibodies were used for detection with Odyssey system (Li-Cor) or western lighting plus-ECL system (PerkinElmer), respectively.

### Protein isolation by Ni-NTA agarose, FLAG M2 affinity gel and anti-HA-agarose

Cell extracts were prepared in PBS containing 1% NP-40 and complete protease inhibitor cocktail (Roche). Ni-NTA agarose beads (Qiagen) were then added to cell extracts and rotated at room temperature for 2 hours. Precipitates were washed three times with PBS containing 1% NP-40, 10 mM and 40 mM imidazole, and then eluted by 350 mM and 500 mM imidazole, respectively. For FLAG M2 affinity gel (Sigma-aldrich) purification, protein samples were incubated with FLAG M2 affinity gel overnight, then the beads were washed by PBS containing 1% NP-40 and 1mM PMSF for 3 times and then boiled in 1 × SDS-PAGE sample loading buffer. For certain experiments, the proteins binding to FLAG affinity gel were eluted by FLAG peptides (150 μg/mL) for further study. For protein immunoprecipitation by anti-HA-agarose, protein samples were incubated with anti-HA-agarose (Sigma-Aldrich) overnight, then the beads were washed by PBS containing 1% NP-40 and 1mM PMSF for 3 times and then boiled in 1 × SDS-PAGE sample loading buffer.

### Deubiquitylation assays

GST-UBP41 was purified from E. coli by glutathione Sepharose beads (GE Healthcare). Purified Ubiquitin conjugates were incubated with GST-UBP41 in 20 mM Tris HCl buffer (pH 8.0) containing 150 mM NaCl, 1 mM DTT, 5 mM MgCl_2_ and 1 mM PMSF at 37 °C for 30 min. The samples were boiled with sample loading buffer and subjected to SDS-PAGE and western blotting analysis.

## Additional Information

**How to cite this article**: Fan, J.-B. *et al*. Identification and characterization of a novel ISG15-ubiquitin mixed chain and its role in regulating protein homeostasis. *Sci. Rep*. **5**, 12704; doi: 10.1038/srep12704 (2015).

## Supplementary Material

Supplementary Information
