# Supplementary material for: Identification and characterization of a novel ISG15-ubiquitin mixed chain and its role in regulating protein homeostasis
Source: Sci Rep. 2015 Jul 30;5:12704. doi: 10.1038/srep12704 (PMC4520236; doi:10.1038/srep12704)
Supplement: Supplementary Information [file srep12704-s1.doc]

**Supplemental information**

**Identification and characterization of a novel ISG15-ubiquitin mixed chain and its role in regulating protein homeostasis**

Jun-Bao Fan^1^, Kei-ichiro Arimoto^1^, Khatereh Motamedchaboki^2^, Ming Yan^1^, Dieter A. Wolf^2^’^3,4^, Dong-Er Zhang^1,5^

^1^Moores UCSD Cancer Center, University of California San Diego, La Jolla CA, 92093, USA

^2^NCI Cancer Center Proteomics Facility, Sanford-Burnham Medical Research Institute, La Jolla, CA 92037

^3^Tumor Initiation & Maintenance Program, Sanford-Burnham Medical Research Institute, 10901 North Torrey Pines Road, La Jolla, CA 92037, USA

^4^San Diego Center for Systems Biology, La Jolla, CA 92093-0375, USA

^5^Department of Pathology and Division of Biological Sciences, University of California San Diego, La Jolla CA, 92093, USA

Corresponding author:

Dr. Dong-Er Zhang

Mail Drop 0815

University of California San Diego

3855 Health Sciences Drive

La Jolla, CA 92093, USA

Email: d7zhang@ucsd.edu

**Supplemental Figures**

**Figure S1. Ub-HA is a substrate of ISG15.** (A) 293T cells were transfected with different constructs as indicated. Cell extracts were immunoprecipitated by anti-HA-Agarose. Proteins binding on the beads were subjected to SDS-PAGE, electroblotted and detected by antibodies as indicated. (B) Generation of HERC5 knocking down cells. 293T cells were transfected with HERC5 shRNA and the stable transfectants were selected by 1 μg/mL puromycin. The expression of HERC5 was measured by RT-qPCR. The mRNA levels in cells expressing control shRNA were set as “1” and the relative mRNA levels in other conditions were normalized by those in control cells. Results were shown as mean + SD from two independent experiments.

**Figure S2. UBB+1 is a substrate of ISG15 in both 293T and HeLa cells.**

(A) ISG15 conjugated to UBB+1 in 293T cells. 293T cells were transfected with different constructs as indicated. Cell lysates were subjected to SDS-PAGE, electroblotted and detected by antibodies as indicated. For WT UBB+1 and lysine deficient form (K0), 0.5 μg DNA was used.

(B) ISG15 conjugated to UBB+1 in HeLa cells. HeLa cells were transfected with different constructs as indicated, cell lysates were subjected to SDS-PAGE, electroblotted and detected by UBB+1 antibody and ISG15 antibody. For UBB+1 (WT), 0.5 μg (+) and 1 μg (++) was used respectively. (C) UBB+1 is a substrate of endogenous ISG15 in MEFs. Cellular ISGylated proteins were immunoprecipitated with Armenian hamster ISG15 monoclonal antibody from MEF cell extracts. ISGylated UBB+1 and cellular ISGylated proteins were detected with UBB+1 antibody and rabbit anti-mouse ISG15 polyclonal antibody.

**Figure S3. Identification of the ISG15 modification site on ubiquitin.** ISG15 modified ubiquitin was purified and analyzed by SDS-PAGE (Coomassie blue staining). After Lys-C Endoproteinase digestion, the expected peptide fragment, which has a modification of ubiquitin by ISG15 at Lys 29, was shown in red.

**Figure S4. ISG15 conjugated to a model ubiquitylated protein.** (A) ISG15 can not be trapped on UBC13 mutant. 293T cells were transfected with different constructs as indicated, cell lysates were subjected to SDS-PAGE and electroblotted and detected by FLAG antibody and ISG15 antibody, respectively. (B) UBC13 double mutant form C87SK92R showed similar modification to the single mutant form UBC13 C87S.

**Figure S5. Characterization of the UBC13, ubiquitin and ISG15 triplex by Mass spectrometry.** Lane 1, samples after FLAG affinity gel purification from 40 mM imidazole washing fraction. Lane 2 and lane 3, samples after FLAG affinity gel purification from 350 mM imidazole eluted fraction. The identified peptides matched with UBC13, ubiquitin, and ISG15 are shown in red.

**Supplemental material and methods**

**In-gel digestion of ISG15 modified ubiquitin and sample preparation for LC-MS/MS Analysis**

To identify modification sites, ISG15 modified ubiquitin was purified by anti-HA-agarose beads and isolated by SDS-PAGE gels. Coomassie blue stained gels were de-stained and then reduced and alkylated by final concentration of 50 mM DTT and 50 mM iodoacetamide prior to digestion by MS Grade Lys-C Endoproteinase (Thermo Fisher Scientific) at a final concentration of 25 ng/µl in 50 mM ammonium bicarbonate for 1 hr on ice and additionally 16 hrs at 37°C using a shaking incubator to assure complete digestion. Digested Lys-C peptides were extracted from gels and transferred to a new tube by following elution process; 100 µl of water added to the gels, sonicated 10 min in water bath and then followed by 1 time in 5% formic acid in water and 4 time extraction by 50% acetonitrile in 5% formic acid in water, once in 70% acetonitrile and last in 100% acetonitrile. All extracted peptides were pooled together and were vacuum dried and re-dissolved in 20 µl of 0.1% Trifluoroacetic acid (TFA). Digested peptides were then concentrated and desalted using a C18 Zip Tip (Millipore). The eluent were then vacuum dried and re-dissolved in 50 µl of LC/MS loading buffer (2% acetonitrile in 0.1% formic acid in water).

**Identification of ubiquitin Lys 29 as the site of ISG15 modification by 1D LC-MS/MS**

Fifty µl of Lys-C digested samples were analyzed by high-resolution, high-accuracy LC-MS/MS, consisting of a Michrom HPLC, a Zorbax C18 peptide trap column (Agilent technologies), a 15 cm Michrom Magic C18 column, a low-flow ADVANCED Michrom MS source, and a LTQ-Orbitrap XL (Thermo Fisher Scientific) mass spectrometer. A 120-min gradient of 10–30% B (0.1% formic acid, 100% acetonitrile) was used to separate the peptides. The total LC time was 160 min. The LTQ-Orbitrap XL was set to scan precursors in the Orbitrap followed by data-dependent MS/MS of the top 10 precursors. The LC-MS/MS raw data were submitted to Sorcerer Enterprise v.3.5 release (Sage-N Research Inc.) with SEQUEST algorithm version 4.0.4 as the search program for peptide/protein identification. SEQUEST was set up to search the target-decoy [ipi.Mouse.V3.73](http://10.1.47.17/sorcerer/databases/view/217) database with the ubiquitin-ISG15 sequence manually indexed (Figure S3) similar to the ChopNSpice method [1]. The database was indexed with the No Enzyme option and searches were done using a precursor mass tolerance of 50 ppm. Differential modifications included 16 Da for methionine oxidation and 57 Da for cysteine carboxyamidomethylation with a maximum of 4 posttranslational modifications per peptide allowed. The search results were viewed, sorted, filtered, and statically analyzed by Peptide/Protein prophet v.4.6.1 (Institute of Systems Biology, Seattle). The minimum Trans-Proteomic Pipeline (TPP) probability score for proteins was set to 0.9, resulting in an FDR < 1%.

**In gel digestion of UBC13-ubiquitin-ISG15 triplex and LC-MS-MS analysis**

**Sample preparation:** To confirm the triple complex formation, UBC13-ubiquitin-ISG15 was purified by Ni-NTA Agarose and FLAG M2 affinity gel and further exercised as described previously [2]. Protein samples were exercised from SDS-PAGE gel and diluted in TNE buffer (50 mM Tris pH 8.0, 100 mM NaCl, 1 mM EDTA). RapiGest SF reagent (Waters Corp.) was added to the mix to a final concentration of 0.1% and samples were boiled for 5 min. TCEP (Tris (2-carboxyethyl) phosphine) was added to 1 mM (final concentration) and the samples were incubated at 37°C for 30 min. Subsequently, the samples were carboxymethylated with 0.5 mg/ml of iodoacetamide for 30 min at 37°C followed by neutralization with 2 mM TCEP (final concentration). Proteins samples prepared as above were digested with trypsin (trypsin:protein ratio - 1:50) overnight at 37°C. RapiGest was degraded and removed by treating the samples with 250 mM HCl at 37°C for 1 h followed by centrifugation at 14000 rpm for 30 min at 4°C. The soluble fraction was then added to a new tube and the peptides were extracted and desalted using Aspire RP30 desalting columns (Thermo Scientific).

**LC-MS-MS**: Trypsin-digested peptides were analyzed by high pressure liquid chromatography (HPLC) coupled with tandem mass spectroscopy (LC-MS/MS) using nano-spray ionization [3]. The nanospray ionization experiments were performed using a TripleTof 5600 hybrid mass spectrometer (ABSCIEX) interfaced with nano-scale reversed-phase HPLC (Tempo) using a 10 cm-100 micron ID glass capillary packed with 5-µm C18 Zorbax^TM^ beads (Agilent Technologies, Santa Clara, CA).  Peptides were eluted from the C18 column into the mass spectrometer using a linear gradient (5–60%) of ACN (Acetonitrile) at a flow rate of 250 μl/min for 1 hr. The buffers used to create the ACN gradient were: Buffer A (98% H_2_O, 2% ACN, 0.2% formic acid, and 0.005% TFA) and Buffer B (100% ACN, 0.2% formic acid, and 0.005% TFA). MS/MS data were acquired in a data-dependent manner in which the MS1 data was acquired for 250 ms at m/z of 400 to 1250 Da and the MS/MS data was acquired from m/z of 50 to 2,000 Da. For independent data acquisition (IDA) parameters MS1-TOF 250 milliseconds, followed by 50 MS2 events of 25 milliseconds each. The IDA criteria, over 200 counts threshold, charge state +2-4 with 4 seconds exclusion. Finally, the collected data were analyzed using MASCOT® (Matrix Sciences) and Protein Pilot 4.0 (ABSCIEX) for peptide identifications.

**RNA Extraction and Analysis**

Real-time RT-PCR was used to examine transcriptional changes of HERC5. Total cellular mRNA was extracted by TRIzol reagent (Gibco-BRL, Invitrogen Corp., Carlsbad, CA) according to the manufacturer’s instructions. First-strand cDNA was synthesized from 1 μg total RNA in a 20-μl reaction volume. RNA expression levels were determined by real-time quantitative PCR (RT-qPCR) using SYBR Green (Kapa Biosystems). With 1μl of cDNA template, PCR amplifications were performed for 45 cycles in a volume of 20μl as follows: denaturating 15 seconds at 95°C, annealing 30 seconds at 60°C, and extension 30 seconds at 60°C. Reactions were performed using Bio-Rad iCycler (Bio-Rad Laboratories) programmed for 45 cycles with quantification and melt curve analysis using iCycler IQ software. Products were further analyzed by agarose gel electrophoresis to confirm the size of products and the absence of primer dimers. Primers for HERC5 used in real-time PCR analysis were as follows: sense primer, 5'-GATTGCTGGAGGGAATCAAA-3'; antisense primer, 5'-TTGGATTTCCCTTTTTGTGC-3'. Transcript levels of the genes of interest were quantitated by Δ-Δ threshold cycle (Ct) method, using the house keeping gene GAPDH for normalization (sense primer, 5’-TCGCTCAGACACCATGGGGAAG-3’; antisense primer, 5’-GCCTTGACGGTGCCATGGAATTTG-3’).

**Supplemental References**

1. Hsiao HH, Meulmeester E, Frank BT, Melchior F, Urlaub H (2009) "ChopNSpice," a mass spectrometric approach that allows identification of endogenous small ubiquitin-like modifier-conjugated peptides. *Mol Cell Proteomics* **8:** 2664-2675

2. Guttman M, Betts GN, Barnes H, Ghassemian M, van der Geer P, Komives EA (2009) Interactions of the NPXY microdomains of the low density lipoprotein receptor-related protein 1. *Proteomics* **9:** 5016-5028

3. McCormack AL, Schieltz DM, Goode B, Yang S, Barnes G, Drubin D, Yates JR, 3rd (1997) Direct analysis and identification of proteins in mixtures by LC/MS/MS and database searching at the low-femtomole level. *Anal Chem* **69:** 767-776
